# A Plea for the Handmaiden

**Published:** 1864

**Authors:** Edward Parrish


### A PLEA FOR THE HANDMAIDEN.

By EDWARD PARRISH.

We often hear Pharmacy represented as the *handmaid of medicine*, and acting on this idea some of our titled colleagues of the Medical Profession, *par excellence*, would exclude the Pharmaceutist from the great temple of medicine, or if they would vouchsafe him an entrance at all, would shut him out in the servant's hall or the scullery. On what grounds this superiority of the Doctors is founded, we may perhaps profitably inquire; if we go to the past we shall find that the Pharmaceutists of to-day, equally with the Physicians, represent the ancient votaries of Æsculapius. If it be true, as we are told, that Hippocrates and Galen, with not a few of their eminent disciples and followers, dispensed their compounds, many of them keeping open shops, while all were perhaps more concerned with Materia Medica and Pharmacy than with either anatomy, physiology, pathology, or surgery, albeit this latter pertained chiefly to the *barber*, who still represents by his trade insignia the ancient blood-letting propensities of the craft, may we not claim at least as ancient and honorable an origin as any branch of the healing art? Measured by the standard of the *present*, we must indeed own to being occupied with the ignoble pursuits of business; we soil our hands with labor, and even demean ourselves with the insignia of self-seeking trade; yet we do produce something wherewith to benefit mankind, and is not the producer, at least, the true hero of this nineteenth century? What would medical art be now, but for the Scientific Pharmacy which evolved Morphia and Quinia, Ferrum redactum, and the Valerianates, and which has added to our new Pharmacopœa, despite the conservatism which controlled its authors, one hundred and eleven new preparations, for the amelioration of suffering and the cure of disease?

These reflections have passed through my mind in coming over some of the flagrant abuses which distinguish the conduct of physicians in our large cities towards their co-laborers, the Pharmaceutists. It is a common observation, that those practitioners who move in what are called "aristocratic circles," and who pander to the follies of the fashionable life, are most addicted to disregarding the recognized amenities of professional intercourse, especially where their humble compeers, the Pharmaceutists, are concerned. Inflated with ideas of their influence and power, and fortified by the greatness of their fees, these professional nabobs delight in patronizing some one renegade Pharmaceutist, who, by the well applied arts of the courtier, ministers to their vanity, while a delicately administered *douceur* occasionally testifies a grateful appreciation of the patronage bestowed. Some, more honest than the rest perhaps, habitually resort to a single dispensing establishment, because they really are persuaded that their prescriptions are better dispensed than at the numerous shops of respectable graduates in Pharmacy, who stand unimpeached, either in the matter of honesty or skill. One of the greatest defects in the education of professional men is, that for want of that contact with men which a business education in early life affords, they so often do not know how to estimate the pretentions of those who lay claim to superior knowledge or skill—to use a common phrase, they are *gullible.* This trait is conspicuous in certain clergymen, who are ready, on the strength of a single apparent cure, to give their influence in favor of the pretentions of some unprincipled quack, whose groundless assumptions would at once vanish into thin air before the steady light of common sense. In these physicians it is observable in the willing credence they give to the extraordinary assertion of the pharmaceutical cicerone, to whose guidance they have willingly lent themselves in their dubious course through the labarinths of Materia Medica; meanwhile, the knowing ones indulge a feeling between indignation and contempt for the practitioner who is so easily led by the nose, and pity for the patients who are the victims of his infatuations. When we are "hectored" by our medical friends because some sufferer has been relieved of a cold or a colic by a timely dose administered "over the counter," without having paid a fee to some one entitled to exact it, we may point him to the numerous graduates of medicine, who have an office adjoining some corner shop belonging to them, where their prescriptions are compounded by a so-called apprentice or clerk, who is paid, perhaps less than a stevedore on the wharf, and whose instructions are, to add the doctor's fee to the cost of the medicine, whenever practical. Or we may direct the attention of our medical complainers to more prominent physicians, who send their prescriptions to a certain store in the neighborhood, the depository of their private receipts, and recommended by no single merit over near and more respectable dispensing stores.

If a poor sufferer comes into my shop asking relief from the pangs of toothache, I feel no hesitation in relieving him if I can, and indeed few actsof my daily routine give me more satisfaction. For this I was never assailed by the nearest dentist with the charge of having interfered with his perogative. Neither, on the same grounds, do I hold myself accountable to the medical faculty for exercising so much humanity and common sense as will help out a suffering fellow-mortal, without resort to the complexities of his diagnosis, prognosis, and other technicalities.

Let me not be charged with hostility to the medical profession. My earliest recollections and life-long associations have taught me to love and honor the high-minded physician who, with zeal for both science and humanity, devotes his life to the most laborious and responsible of pursuits; but this very respect for the physician as he *should* be, induces me to place a proper estimate upon the physician as he too often *is*, and to protest, in the name of common honesty and fair dealing, against the unprofessional favoritism to which I have alluded as being notorious, especially in our large cities. And now, on entering the second decade in the history of this Association, let me assert for American Pharmacy the claim, founded on a common origin and kindred objects, to an equal and independent place, no longer as a handmaiden, but as a modest and docile sister, beside the more numerous and distinguished branch of the medical family. May we all strive to *deserve* such a position.—*From Proceedings of the American Pharmaceutical Association.*

